# Salophene
**Pharm. Zeit.*, 1891, p. 678; *Pharm. Post*, No. 51, 1891.


**Published:** 1892-03-19

**Authors:** 


### THERAPEUTICS.

### SALOPHENE.*

Salophene is a derivative of salol. In the phenyl group of the ether of phenyl salicylic acid an atom of hydrogen is replaced by the group acetylamide (in the *para* position). Salophene is obtained by treating para-nitrophenol by salicylic acid, reducing the nitrophenol by zinc and hydrochloric acid in an amide. Salophene contains approximately as much as fifty per cent. of salicylic acid, and forms small scales, tasteless and without smell, with a neutral reaction. Almost insoluble in cold water, and only slightly soluble in hot water, it is most conveniently dissolved in alcohol or ether. By the addition of a caustic alkali, salophene is readily dissolved, even in the cold. In the test tube, in the presence of alkalies, and also in the body, salophene is split up into salicylic acid and into acetyl-para-amidophénol; these substances pass off by the urine, and can be readily detected by suitable re-agent. Owing to the presence of the amido-phenol group, salophene is less toxic than salol. According to Guttman, salophene should prove an excellent remedy in acute polyarticular rheumatism. The dose is four to six grains daily, in pills, or in the form of compressed tablets.

